# Functionality of hybrid nanofluid with microorganisms influence inside absorptive channel subject to magnetic field and heat source

**DOI:** 10.1186/s11671-026-04721-4

**Published:** 2026-06-24

**Authors:** Adnan, Khaleeq ur Rehman, Marwa Ben Slimene, Mohamed Arbi Khlifi, Sami Ullah Khan, Rashed O. Abu Hammour, Seham M. Al-Mekhlafi, Amr Sayed Hassan Abdallah, Mohamed Bechir Ben Hamida

**Affiliations:** 1 Department of Mathematics, Mohi-ud-Din Islamic University, Nerian Sharif, Trarkhel, AJ&K 12080 Pakistan; 2 Department of Computer Engineering, College of Computer science and Engineering, University of Ha’il, P.O. Box 2440, Ha’il City, Saudi Arabia; 3 Department of Electrical Engineering, Faculty of Engineering, Islamic University of Madinah, 42351 Madinah, Saudi Arabia; 4 Department of Mathematics, Namal University, Mianwali, 42250 Pakistan; 5 Faculty of Technical Education, Hourani Center for Applied Scientific Research (HCASR), Al-Ahliyya Amman University, Amman, Jordan; 6 Mathematics Department, Faculty of Education, Sana’a University, Sana’a, Yemen; 7 Department of Architectural Engineering, College of Engineering, Imam Mohammad Ibn Saud Islamic University (IMSIU), 11432 Riyadh, Saudi Arabia; 8 Deanship of Scientific Research, Imam Mohammad Ibn Saud Islamic University (IMSIU), Riyadh, Saudi Arabia

**Keywords:** Hybrid nanofluid, Heat transfer, Microorganisms’ nanoparticles, VIM, Permeable media, Heat generation/absorption radiation

## Abstract

Investigation of nanofluids under microbial movement is a potential topic from applications point of view. This study focuses on the analysis of hybrid nanomaterial based microorganism model between finite boundaries. These boundaries consist of two walls having expansion or contraction property. Formulation of the problem comprises governing laws representing the flow of microorganism in the presence of physical effects and hybrid nanofluid effective characteristics. Similarity functions are adopted for conversion of the primary model into final form and then studied through Variational Iteration scheme. Successful implementation of the scheme provided considerable convergence and better results for the model with fluctuating physical parameters. It is found that increasing permeation and magnetic field significantly controlled the velocity in the channel. Presence of heat generation influentially enhances the temperature for both expansion and contraction cases; while the Soret effects help to augment the density motile of microorganism. The shear drag varies from 0.9057 to 1.1458 ($\:{\phi\:}_{1}$), 0.9076 to 0.2470 ($\:S$) in contraction case at the lower surface. This magnitude changes from 0.6762 to 0.6774 ($\:{\phi\:}_{1}$), 0.6793 to 0.1558 ($\:S$) at upper expanding surface. Contracting of the walls promoting this factor from 1.9017 to 1.8970 and 1.3982 to 1.3964, for $\:{\phi\:}_{1}$ and $\:S$, respectively. Further, the expanding walls heat transfer rate improves from 0.0509 to 0.0568, 1.4957 to 1.4981 and it depreciates from 0.0480 to 0.0461, and 1.4922 to 1.4839 in contracting walls.

## Introduction

Study of microorganism model subject to the different physical controls is fascinating research area. Devekar et al. [1] examined the unsteady micropolar fluid flow in infinite plate geometry with one end of plates is set to be moveable and the other is fixed at in rest situation. The Laplace transform domain (LTD) is implemented to acquire the solutions for microrotation and fluid velocity. Also, various material parameters discussed and observed their key role the model dynamics. Ojjela et al. [2] presented the heat transfer under MHD effects on microorganism’s fluid dynamic under electrically conducting condition between parallel porous surfaces which is caused by periodic suction or injection at plates. The Quasilinearization scheme applied on the system and studied the velocity and microrotation distribution for different geometric and microorganism fluid’s conditions. Furthermore, the temperature is examined to be increased through time interval. Slayi et al. [3] investigated microorganism flow behaviour under the physical conditions and obtained the results. Numerical outcomes of local thermal gradient and shear drag are also discussed through numerical computation.

Bhatti et al. [4] described the micro-nanofluid on stretching porous sheet. The tabulated evaluation has also been offered for shear drag as a superior case for unified study. Hsiao et al. [5] investigated hydromagnetic and micropolar nanofluids flow and thermal analysis of energy management problem with heat transfer of under magnetic and dissipation properties towards a stretching sheet. The finite-difference method (FDM) with similarity transformation has been analyzed for micropolar flow and pronounced that the temperature is declined as the Prandtl number accumulative, and improved as the Eckert number increases. Alizadeh et al. [6] examined the impacts of MHD on microbe’s based fluid flow and heat transfer by considering thermal radiation influence and apply DRA scheme on microbe’s model with validation of method. The characteristics of diverse parameters explored and outputs state that augment of the micro parameter results in slightly increases of concentration profile. Khan et al. [7] studied 3D microorganism’s nanofluid flow in a rotating coordinate under Darcy Forchheimer properties between horizontal plates.

Influence of chemical reactive species [8] together with buoyancy effects has been discussed by Javaid et al. [9]. The results deduced that the transfer of heat is in inverse relation with Prandtl number while Eyring Powell number is useful to boosts the temperature. Waseem et al. [10] experienced the Cattaneo Christove effects through viscoelastic fluid with moving organisms at micropolar [11] level. The study pointed that the findings would be useful for industrial applications where transport of heat is a potential issue. Further, results investigated through graphical illustrations with physical ranges of the quantities. Another analysis unveiling the role of multiple constraints with interacting microorganism is reported in [12]. It is shown that increasing porosity and magnetic influence decline the velocity. Further, recent remarkable investigations regarding the applications of nanofluids subject to the important quantities are demonstrated in the Refs. [13–17], and [18]. These studies discussed multiple ways of engineering applications of nanofluids [19], under different circumstances.

Waini et al. [20] studied steady flow of hybrid nanofluid past absorptive stretching/shrinking region. It is noticed that the heat transmission improved for hybrid nanoliquid. Dash et al. [21] reported the study of micropolar steady incompressible fluid flow with electrically conducting in plates along first order biochemical antiphon, heat compress, source /absorption. The non-dimensional ODEs [22] are acquired by the support of correspondence conversions and then studied numerically. Recently, the trends towards the combination of non-Newtonian fluids with nanomaterials have been observed. Wang et al. [23] conducted deep study of Casson fluid with addition of hybridity of three types of particles. The output shows major contribution of these particles in the heat mechanism and suggested their application areas. Rasool et al. [24] noticed that activation energy is a major parameter which magnify the concentration while chemical reaction controls it. Sajid et al. [25] explored the features of two phase nanofluid problem subject to slip and radiation effects. The problem formulated for convective surface and found considerable increase in the heat due to strong convection and radiation factor. Shahzad et al. [26], and Wang et al. [27] introduced models for bioconvection under CCR and magnetic control, and Carreau entropy model using Keller Box scheme, respectively. The findings of the studies provided influential physical results regarding the heat, momentum and mass transfer in bioconvection.

Dawar et al. [28] discussed an MHD based model with slip effects. The thermal field reinforces with increasing factors Biot number, non-uniform sink/source of heat, temperature ratio aspect and thermal emission. Khan et al. [29] studied the effects of microparticles on the problem results under varying constraints. The mathematical grades showed, an accumulation in the magnetic parameter is response to diminishing in Nusselt number, or an aggregation in shear drag, couple stress at the surface. Kumar et al. [30] examined stokes flow of unsteady and incompressible micropolar fluid among two absorbent plates. The geometry is exposed to periodic suction/ periodic injection which are execute at the lower/upper plate respectively. Deviations of velocity are revealed explicitly and then obtained Stream function.

Alsoufi et al. [31] performed ANN analysis of engine oil with consistent dispersion of ternary nanomaterial. The problem designed for magnet based surface along with physical conditions. The obtained results discussed deeply in the view of physical facts behind them and also suggested potential application areas. Investigation related to hetro and homogenous reactions and their role in the heat mechanism of Mawell fluid has been conducted by Gangadhar et al. [32]. They used regularization based NN scheme and found the output with excellent accuracy. The findings shown appropriate ranges of the constraints for better results over the domain. Sarma et al. [33] reported the role of heat generation on hemodynamic Carreau model in cylindrical configuration. The findings reveal that Carreau fluid with blood and nanoparticles have fascinating impacts on the heat and momentum transfer and these would be of interest in biomedical engineering. The studies that convinced towards the use of nanoparticles for medication and blood circulation and morphology contribution on the heat mechanism have been demonstrated in the Refs. [34], and [35], respectively. These investigations highlighted the importance of nanomaterials and their shapes for improved thermal applications [36], in various applied fields.

Rafique et al. [37] studied the Buongiorno’s model with Brownian, thermal conductivity and thermophoretic effects taken into account in stretchable sheet. Compatible conversions are executed to simplified modeled PDE’s and are treated by numerically. Assumption strained that energy transfer rates augment against addition in Soret factor while shear drag moderates. Tayebi et al. [38] examined micropolar Al_2_O_3_-water nanofluid flow under thermal buoyancy convection and heat exchange. The numerical FEM (finite element method) approach was applied to system that govern the flow occurrence. The rate of heat interchange intensifies as the *R*_*a*_ and *A*_*r*_ parameters increased but declines when the increasing *K*. Khan et al. [39] discussed the behaviour of microorganism fluid in parallel geometry. The heat exchange rates, entropy production were indomitable for varied limits of Rayleigh, enclosure inclination angle (*χ*), vortex viscosity and geometric ratio (*A*_*R*_) parameters. Recent investigations on the promising application of fluids and their coupling with variety of nanoparticles and physical quantities have been discussed by various researchers in the Refs. [40–45].

Previous studies on related to bioconvection in expanding and contracting channel have basically focused on simple organism model, ignoring the joint contribution of heat generation, magnetic field, and cross diffusion mechanism. Most of the reported investigations have been reported using semi analytical of numerical techniques rather than iterative scheme. Although, the recent studies have presented hybrid type nanofluids even with analytical schemes but they infrequently considered microbial movement with influence of these mentioned parameters in deformable channel. Thus, there remains a potential research gap in developing a model that combine hybrid nanofluid, multi interacting physical quantities and bioconvection in deformable channel. This attempt would be useful for biomedical applications. Further, the study presents this need by developing such a dynamic model using VIM for efficient output. It will offer a vibrant framework to analyze the interaction between microspecies and physical constraints.

This research has the following objectives.

- To develop a fascinating framework for microorganism model in the channel with walls that are capability of expansion and contraction.
- To modify the problem for heat and mass transfer with heat source and magnetic field.
- To discuss the role of cross diffusion and nanoparticles concentration on the dynamics of microorganism’s movement and heat transfer.
- To analyzed the comparative skin friction and heat transfer rate simultaneously under changes in the parameters.

## Model development

**Fig. 1:**
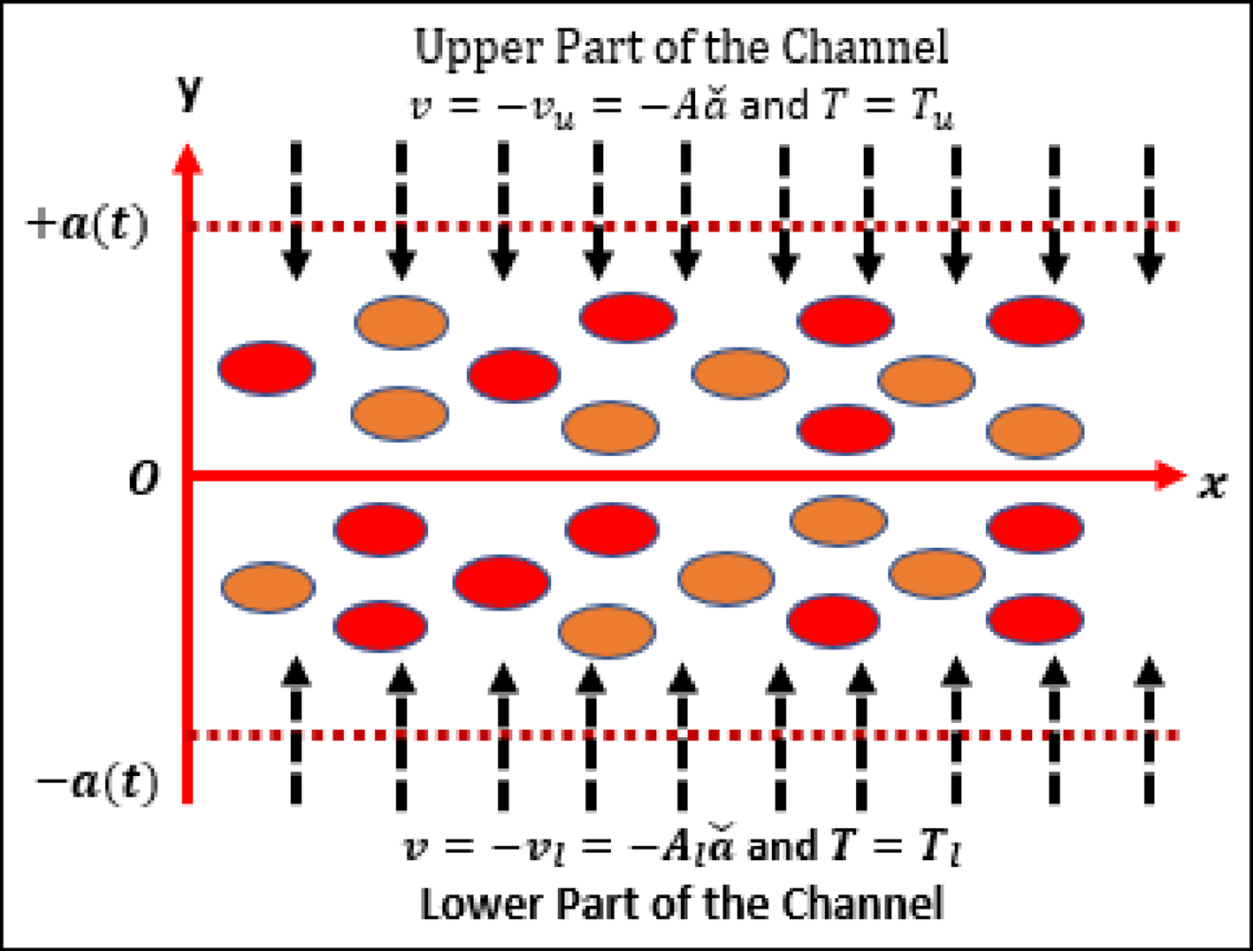
Configuration of the problem

Consider 2D magnetically conducting microorganism nanofluid flow among two plates that are parallel and horizontal. Also, positioned of plates at $\:a\left(t\right)$ and $\:-a\left(t\right)$ along y-axis. It is presumed that the $\:x$ has in *u* and $\:y$ has in *v* directions. The Darcy effect and heat generation exists in the channel with permeable walls. The fluid flows along $\:x-axis$ with normal magnetic field. Configuration of the problem is furnished in Fig. 1.

### Assumptions

The fluid is incompressible and unsteady between uniformly expanding and contracting walls. Hybrid nanomaterial uniformly saturated in microorganism based fluid. Further, the direction of imposed magnetic field is normal to the channel and the model is reliable in the laminar regime.

In the view of assumptions, the leading model of micropolar nanofluid is given in the following form [46]:

1 $$\:\frac{\partial\:\stackrel{\sim}{u}}{\partial\:x}+\frac{\partial\:\stackrel{\sim}{v}}{\partial\:y}=0$$

2 $$\:{\rho\:}_{hnf}\left(\:\frac{\partial\:\stackrel{\sim}{u}}{\partial\:t}+\stackrel{\sim}{u}\frac{\partial\:\stackrel{\sim}{u}}{\partial\:\stackrel{\sim}{x}}+\stackrel{\sim}{v}\frac{\partial\:\stackrel{\sim}{u}}{\partial\:\stackrel{\sim}{y}}\:\right)=-\frac{\partial\:\stackrel{\sim}{P}}{\partial\:\stackrel{\sim}{x}}+{\mu\:}_{hnf}\left[\:\frac{{\partial\:}^{2}\stackrel{\sim}{u}}{\partial\:{\stackrel{\sim}{x}}^{2}}\:\:+\frac{{\partial\:}^{2}\stackrel{\sim}{u}}{\partial\:{\stackrel{\sim}{y}}^{2}}\:\right]-{\sigma\:}_{hnf}{{B}_{0}}^{2}\stackrel{\sim}{u}-\frac{{k}_{hnf}{\stackrel{\sim}{u}}^{2}}{k{\rho\:}_{hnf}}$$

3 $$\:{\rho\:}_{hnf}\left(\:\frac{\partial\:\stackrel{\sim}{v}}{\partial\:t}+\stackrel{\sim}{u}\frac{\partial\:\stackrel{\sim}{v}}{\partial\:\stackrel{\sim}{x}}+\stackrel{\sim}{v}\frac{\partial\:\stackrel{\sim}{v}}{\partial\:\stackrel{\sim}{y}}\:\right)=-\frac{\partial\:\stackrel{\sim}{P}}{\partial\:\stackrel{\sim}{y}}+{\mu\:}_{hnf}\left[\:\frac{{\partial\:}^{2}\stackrel{\sim}{v}}{\partial\:{\stackrel{\sim}{x}}^{2}}\:\:+\frac{{\partial\:}^{2}\stackrel{\sim}{v}}{\partial\:{\stackrel{\sim}{y}}^{2}}\:\right]-{\sigma\:}_{hnf}{{B}_{0}}^{2}\stackrel{\sim}{v}-\frac{{k}_{hnf}{\stackrel{\sim}{v}}^{2}}{k{\rho\:}_{hnf}}$$

4 $$\:{\left(\rho\:{C}_{p}\right)}_{hnf}\left(\:\frac{\partial\:\stackrel{\sim}{T}}{\partial\:t}+\stackrel{\sim}{u}\frac{\partial\:\stackrel{\sim}{T}}{\partial\:\stackrel{\sim}{x}}+\stackrel{\sim}{v}\frac{\partial\:\stackrel{\sim}{T}}{\partial\:\stackrel{\sim}{y}}\:\right)={k}_{hnf}\left[\:\frac{{\partial\:}^{2}\stackrel{\sim}{T}}{\partial\:{\stackrel{\sim}{x}}^{2}}\:\:+\frac{{\partial\:}^{2}\stackrel{\sim}{T}}{\partial\:{\stackrel{\sim}{y}}^{2}}\:\right]+{Q}_{0}\left(\stackrel{\sim}{T}-{\stackrel{\sim}{T}}_{0}\right)+\frac{{D}_{m}{K}_{T}}{{C}_{s}{C}_{p}}\left[\:\frac{{\partial\:}^{2}\stackrel{\sim}{C}}{\partial\:{\stackrel{\sim}{x}}^{2}}\:\:+\frac{{\partial\:}^{2}\stackrel{\sim}{C}}{\partial\:{\stackrel{\sim}{y}}^{2}}\:\right]$$

5 $$\:\left(\:\frac{\partial\:\stackrel{\sim}{C}}{\partial\:t}+\stackrel{\sim}{u}\frac{\partial\:\stackrel{\sim}{C}}{\partial\:\stackrel{\sim}{x}}+\stackrel{\sim}{v}\frac{\partial\:\stackrel{\sim}{C}}{\partial\:\stackrel{\sim}{y}}\:\right)=\frac{{D}_{m}{K}_{T}}{{C}_{s}{C}_{p}}\left[\:\frac{{\partial\:}^{2}\stackrel{\sim}{T}}{\partial\:{\stackrel{\sim}{x}}^{2}}\:\:+\frac{{\partial\:}^{2}\stackrel{\sim}{T}}{\partial\:{\stackrel{\sim}{y}}^{2}}\:\right]+D\left[\:\frac{{\partial\:}^{2}\stackrel{\sim}{C}}{\partial\:{\stackrel{\sim}{x}}^{2}}\:\:+\frac{{\partial\:}^{2}\stackrel{\sim}{C}}{\partial\:{\stackrel{\sim}{y}}^{2}}\:\right]$$

6 $$\:\left(\:\frac{\partial\:\stackrel{\sim}{n}}{\partial\:t}+\stackrel{\sim}{u}\frac{\partial\:n}{\partial\:\stackrel{\sim}{x}}+\stackrel{\sim}{v}\frac{\partial\:\stackrel{\sim}{n}}{\partial\:\stackrel{\sim}{y}}\:\right)+\left(\frac{b{W}_{1}}{{C}_{b}-{C}_{f}}\right)\frac{\partial\:}{\partial\:y}\left(n\frac{\partial\:\stackrel{\sim}{C}}{\partial\:\stackrel{\sim}{y}}\right)={D}_{m}\left[\:\frac{{\partial\:}^{2}\stackrel{\sim}{n}}{\partial\:{\stackrel{\sim}{x}}^{2}}\:\:+\frac{{\partial\:}^{2}\stackrel{\sim}{n}}{\partial\:{\stackrel{\sim}{y}}^{2}}\:\right]$$

The associated model conditions are:

7 $$\:\:\left\{\:\begin{array}{c}\stackrel{\sim}{u}=0,\:T={T}_{l\:},\:\stackrel{\sim}{v}=-{v}_{l}=-h{A}_{l}\:\:\:\:at\:\:\:\stackrel{\sim}{y}=-\:h\left(t\right)\\\:\stackrel{\sim}{u}=0,\:T={T}_{u}\:,\stackrel{\sim}{v}{=-v}_{u}=-h{A}_{u}\:\:\:at\:\:\stackrel{\sim}{y}=+h\left(t\right)\end{array}\right.$$

8 $$\:\chi\:=\frac{\partial\:\stackrel{\sim}{v}}{\partial\:\stackrel{\sim}{x}}-\frac{\partial\:\stackrel{\sim}{u}}{\partial\:\stackrel{\sim}{y}}$$

To remove the pressure influence and essential derivatives yield the following form:

9 $$\:\:{\rho\:}_{nf}(\frac{\partial\:\stackrel{\sim}{\chi\:}}{\partial\:t}+\stackrel{\sim}{u}\frac{\partial\:\stackrel{\sim}{\chi\:}}{\partial\:\stackrel{\sim}{x}}+\stackrel{\sim}{v}\frac{\partial\:\stackrel{\sim}{\chi\:}}{\partial\:\stackrel{\sim}{y}})={\mu\:}_{nf}\left[\frac{{\partial\:}^{2}\stackrel{\sim}{\chi\:}}{\partial\:{\stackrel{\sim}{x}}^{2}}+\frac{{\partial\:}^{2}\stackrel{\sim}{\chi\:}}{\partial\:{\stackrel{\sim}{y}}^{2}}\right]$$

10 $$\:{\rho\:}_{nf}\left({\stackrel{\sim}{u}}_{yt}+\stackrel{\sim}{u}{\stackrel{\sim}{u}}_{yx}+\stackrel{\sim}{v}{\stackrel{\sim}{u}}_{yy}\right)={\mu\:}_{nf}{\stackrel{\sim}{u}}_{yyy}$$

Now, introducing the consequent transform variables for further simplification of our model:

11 $$\:\eta\:=\frac{y}{h}$$

12 $$\:\stackrel{\sim}{u}=\frac{{\upsilon\:}_{f\:\stackrel{\sim}{x}\:{\stackrel{\sim}{f}}_{\eta\:}}}{h},\:\stackrel{\sim}{v}=-\frac{{\upsilon\:}_{f\:\:\stackrel{\sim}{f\:}\left(\eta\:,t\right)}}{{h}^{2}},\:\stackrel{\sim}{\psi\:}=-\frac{{\upsilon\:}_{f\:\stackrel{\sim}{x}\:\stackrel{\sim}{f\:}\left(\eta\:,t\right)}}{h},\:\beta\:\left(\eta\:\right)=\frac{\stackrel{\sim}{T\:}-\:{\stackrel{\sim}{T}}_{u}\:}{{\stackrel{\sim}{T}}_{i}-{\stackrel{\sim}{T}}_{u}},\:\phi\:\left(\eta\:\right)=\frac{\stackrel{\sim}{C\:}-\:{\stackrel{\sim}{C}}_{u}\:}{{\stackrel{\sim}{C}}_{i}-{\stackrel{\sim}{C}}_{u}},\:\gamma\:\left(\eta\:\right)=\frac{n-\:{\stackrel{\sim}{n}}_{u}\:}{{n}_{i}-{n}_{u}}$$

Now, the further assumptions on the previous BCs income the following version.

13 $$\:\left\{\begin{array}{c}{\stackrel{\sim}{f}}_{\eta\:}=0,\stackrel{\sim}{f\:}={{R}_{e}}_{\:,\:\:\:}Re1=\frac{{h{h}^{.}A}_{u}}{{\upsilon\:}_{f}}\:at\:\eta\:=-1\\\:{\stackrel{\sim}{f}}_{\eta\:}=0,\stackrel{\sim}{f}={{R}_{e}}_{\:,\:\:\:}Re1=\frac{{h{h}^{.}A}_{u}}{{\upsilon\:}_{f}}\:at\:\eta\:=1\end{array}\right.$$

Here $\:{R}_{e}$ is associated to the permeation effects which is positive and negative under section/injection. Further, the second set of transformations is enlisted in Eq. (14).

14 $$\:x=\frac{\stackrel{\sim}{x}}{h},u=\frac{\stackrel{\sim}{u}}{h},v=\frac{\stackrel{\sim}{v}}{h},f=\frac{\stackrel{\sim}{f}}{Re1}$$

Thermophysical properties of hybrid nanofluid are defined as below. Further, the individual properties of hybrid nanomaterial (Al_2_O_3_ and CuO) and base fluid are given in the Ref. [47].

15 $$\:\frac{{\rho\:}_{hnf}}{{\rho\:}_{f}}=(1-{\phi\:}_{2})\left(\left(1-{\phi\:}_{1}\right)+\phi\:1\frac{{\rho\:}_{s1}}{{\rho\:}_{f}}\right)+{\phi\:}_{2}\frac{{\rho\:}_{s2}}{{\rho\:}_{f}},$$

16 $$\:\frac{{\left({\rho\:C}_{p}\right)}_{hnf}}{{\left(\rho\:{c}_{p}\right)}_{f}}=(1-{\phi\:}_{2})\left(\left(1-{\phi\:}_{1}\right)+\phi\:1\frac{{\left({\rho\:C}_{p}\right)}_{s1}}{{\left({\rho\:C}_{p}\right)}_{f}}\right)+{\phi\:}_{2}\frac{{\left({\rho\:C}_{p}\right)}_{s2}}{{\left({\rho\:C}_{p}\right)}_{f}},$$

17 $$\:\frac{{\mu\:}_{hnf}}{{\mu\:}_{f}}=\frac{1}{{\left(1-{\phi\:}_{1}\right)}^{2.5}{\left(1-{\phi\:}_{2}\right)}^{2.5}},$$

18 $$\:\left.\begin{array}{c}\frac{{k}_{hnf}}{{\widehat{k}}_{nf}}=\frac{{\widehat{k}}_{{s}_{2}}+2{\widehat{k}}_{nf}-2{\phi\:}_{2}({\widehat{k}}_{nf}-{\widehat{k}}_{{s}_{2}})}{{\widehat{k}}_{{s}_{2}}+2{\widehat{k}}_{nf}+{\phi\:}_{2}({\widehat{k}}_{nf}-{\widehat{k}}_{{s}_{2}})}\\\:\frac{{k}_{hnf}}{{\widehat{k}}_{f}}=\frac{{\widehat{k}}_{{s}_{1}}+2{\widehat{k}}_{f}-2{\phi\:}_{1}({\widehat{k}}_{f}-{\widehat{k}}_{{s}_{1}})}{{\widehat{k}}_{{s}_{1}}+2{\widehat{k}}_{f}+{\phi\:}_{1}({\widehat{k}}_{f}-{\widehat{k}}_{{s}_{1}})}\end{array}\right\},$$

19 $$\:\left.\begin{array}{c}\frac{{\sigma\:}_{hnf}}{{\widehat{\sigma\:}}_{bnf}}=\frac{{\widehat{\sigma\:}}_{{s}_{2}}+2{\widehat{\sigma\:}}_{bnf}-2{\phi\:}_{2}\left({\widehat{\sigma\:}}_{nf}-{\widehat{\sigma\:}}_{{s}_{2}}\right)}{({\widehat{\sigma\:}}_{{s}_{2}}+2{\widehat{\sigma\:}}_{bnf}+{\phi\:}_{2}({\widehat{\sigma\:}}_{nf}-{\widehat{\sigma\:}}_{{s}_{2}})}\\\:\frac{{\sigma\:}_{nf}}{{\widehat{\sigma\:}}_{f}}=\frac{{\widehat{\sigma\:}}_{{s}_{1}}+2{\widehat{\sigma\:}}_{f}-2{\phi\:}_{1}\left({\widehat{\sigma\:}}_{f}-{\widehat{\sigma\:}}_{{s}_{1}}\right)}{({\widehat{\sigma\:}}_{{s}_{1}}+2{\widehat{\sigma\:}}_{f}+{\phi\:}_{1}({\widehat{\sigma\:}}_{f}-{\widehat{\sigma\:}}_{{s}_{1}})}\end{array}\right\},$$

Exercising the above information in the problem, the model transformed in the following form.

20 $$\:{F}^{{\prime\:}{\prime\:}{\prime\:}{\prime\:}}+\frac{\frac{{\rho\:}_{hnf}}{{\rho\:}_{f}}}{\frac{{\mu\:}_{nf}}{{\mu\:}_{f}}}\left(\alpha\:\left(\eta\:{F}^{{\prime\:}{\prime\:}{\prime\:}}+{F}^{{\prime\:}{\prime\:}}\right)+{R}_{e1}\left(F{F}^{{\prime\:}{\prime\:}{\prime\:}}-{F}^{{\prime\:}}{F}^{{\prime\:}{\prime\:}}\right)\right)-{M}^{2}\frac{1}{\frac{{\rho\:}_{hnf}}{{\rho\:}_{f}}}{F}^{{\prime\:}{\prime\:}}=0$$

21 $$\:F\left(-1\right)=S,F\left(1\right)=0,{F}^{{\prime\:}}\left(-1\right)=0,{F}^{{\prime\:}}\left(1\right)=0$$

22 $$\:{\beta\:}^{{\prime\:}{\prime\:}}+\frac{{k}_{f}}{{k}_{hnf}}({\xi\:}^{{\prime\:}{\prime\:}}{P}_{r}{D}_{u}-\frac{{\left({\rho\:C}_{p}\right)}_{hnf}}{{\left({\rho\:C}_{p}\right)}_{f}}\left(\eta\:{\beta\:}^{{\prime\:}}-{R}_{e1}F\right))+\frac{{k}_{f}\beta\:{Q}_{1}}{{k}_{hnf}}=0$$

23 $$\:\beta\:\left(-1\right)=1,\:\beta\:\left(1\right)=0$$

24 $$\:{\xi\:}^{{\prime\:}{\prime\:}}+{S}_{r}{S}_{c}{\beta\:}^{{\prime\:}{\prime\:}}-\left(\:\eta\:+{{S}_{c}R}_{e1}\right){\xi\:}^{{\prime\:}}=0$$

25 $$\:\xi\:\left(-1\right)=1,\:\xi\:\left(1\right)=0$$

26 $$\:{\gamma\:}^{{\prime\:}{\prime\:}}-{P}_{e}\left(\delta\:+\gamma\:\right){\xi\:}^{{\prime\:}{\prime\:}}-{P}_{e}{\gamma\:}^{{\prime\:}}{\xi\:}^{{\prime\:}}-\left(\eta\:+{R}_{e1}{L}_{b}F\right){\gamma\:}^{{\prime\:}}=0$$

27 $$\:\gamma\:\left(-1\right)=1,\gamma\:\left(1\right)=0$$

The quantities of interest are further modeled in the following formulas.

28 $$\:{C}_{F}=\frac{\tau\:a\left(t\right)}{{\widehat{\rho\:}}_{hnf}{v}_{l}^{2}},\:{\tau\:}_{w}={\mu\:}_{hnf}\left[\frac{\partial\:\stackrel{\sim}{u}}{\partial\:y}\right]\:when\:y=\mp\:a$$

29 $$\:{N}_{u}=\frac{a}{{k}_{f}({T}_{l}-{T}_{u})}\left[{k}_{hnf}\frac{\partial\:T}{\partial\:y}\right]\:$$

Using appropriate information, these further simplified as below.

30 $$\:{R}_{l}^{2}{C}_{Fl}=\left(\frac{{\mu\:}_{hnf}}{{\mu\:}_{f}}\right){\left(\frac{{\rho\:}_{hnf}}{{\rho\:}_{f}}\right)}^{-1}F^{\prime\prime\:}(-1)$$

31 $$\:{R}_{u}^{2}{C}_{Fu}=\left(\frac{{\mu\:}_{hnf}}{{\mu\:}_{f}}\right){\left(\frac{{\rho\:}_{hnf}}{{\rho\:}_{f}}\right)}^{-1}F^{\prime\prime\:}\left(1\right)$$

32 $$\:{\stackrel{\sim}{N}}_{ul}=\left|{\beta\:}^{{\prime\:}}\left(-1\right)\right|\frac{{\widehat{k}}_{hnf}}{{\widehat{k}}_{f}}$$

33 $$\:{\stackrel{\sim}{N}}_{up}=\left|{\beta\:}^{{\prime\:}}\left(1\right)\right|\frac{{\widehat{k}}_{hnf}}{{\widehat{k}}_{f}}$$

## Mathematical analysis

The VIM is implemented to achieve the results.

**Step-I**-We introduce the nonlinear, linear and nonhomogeneous factors such as ($\overbrace {{\Re }}\nolimits_{{{01}}} ,\overbrace {{\Re }}\nolimits_{{{02}}}$), ($\overbrace {\aleph }\nolimits_{{01}} ,\overbrace {\aleph }\nolimits_{{02}}$) and ($\overbrace {q}\nolimits_{{01}}^{*} ,\overbrace {q}\nolimits_{{02}}^{*}$) resp. with linear operators ($\overbrace {{\mathcal{L}}}\nolimits_{{01}} ,\overbrace {{\mathcal{L}}}\nolimits_{{02}}$),

$$ \overbrace {{\mathcal{L}}}\nolimits_{{{01}}} F + \overbrace {{\Re }}\nolimits_{{{01}}} F + \overbrace {\aleph }\nolimits_{{{01}}} F + \overbrace {q}\nolimits_{{{01}}}^{*} = 0~\;and\;\overbrace {{\mathcal{L}}}\nolimits_{{{02}}} \gamma + \overbrace {{\Re }}\nolimits_{{{02}}} \gamma + \overbrace {\aleph }\nolimits_{{{02}}} \gamma + \overbrace {q}\nolimits_{{{02}}}^{*} = 0 $$

**Step-II**–Here, describing multipliers called Lagrange multipliers which has same order as model.

$$ \mathop {\lambda_{F}} \limits^{ \vee } = \frac{{\left( { - 1} \right)^{{j*}} \left( {\eta - s} \right)^{{j^{*} - 1}} }}{{\left( {j^{*} - 1} \right)!}}\;{\mathrm{And}}\;\mathop {\lambda_{\gamma }} \limits^{ \vee } = \frac{{\left( { - 1} \right)^{{j*}} \left( {\eta - s} \right)^{{j^{*} - 1}} }}{{\left( {j^{*} - 1} \right)!}} $$

**Step-III**-Choose initial guess/values

$$ \mathop {F_{0}} \limits^{ \vee } = \mathop \sum \limits_{{i = 0}}^{r} \frac{{\eta ^{i} F\left( 0 \right)}}{{i!}}\;~{\mathrm{and}}\;~\mathop {\gamma_{0}} \limits^{ \vee } = \mathop \sum \limits_{{i = 0}}^{r} \frac{{\eta ^{i} \gamma \left( 0 \right)}}{{i!}} $$.

**Step-IV**-Finally, Formula for iterations

$$ F_{{l + 1}} = \mathop {F_{0}}\limits^{ \vee } + \mathop \int \limits_{0}^{\eta } \mathop {\lambda_{F}} \limits^{ \vee } \left( { - \overbrace {{\Re }}\nolimits_{{{01}}} F\left( s \right) - \overbrace {\aleph }\nolimits_{{{01}}} F\left( s \right) - \overbrace {q}\nolimits_{{{01}}}^{*} \left( s \right)} \right)ds,~l \ge 0$$

$$ \gamma _{{l + 1}} = \mathop {\gamma _{0}} \limits^{ \vee } + \mathop \int \limits_{0}^{\eta } \mathop {\lambda_{\beta }} \limits^{ \vee } \left( { - \overbrace {{\Re }}\nolimits_{{2}} \gamma \left( s \right) - \overbrace {\aleph }\nolimits_{{{02}}} \gamma \left( s \right) - \overbrace {q}\nolimits_{{{02}}}^{*} \left( s \right)} \right)ds,~l \ge 0 $$.

## Discussion of results

**Fig. 2:**
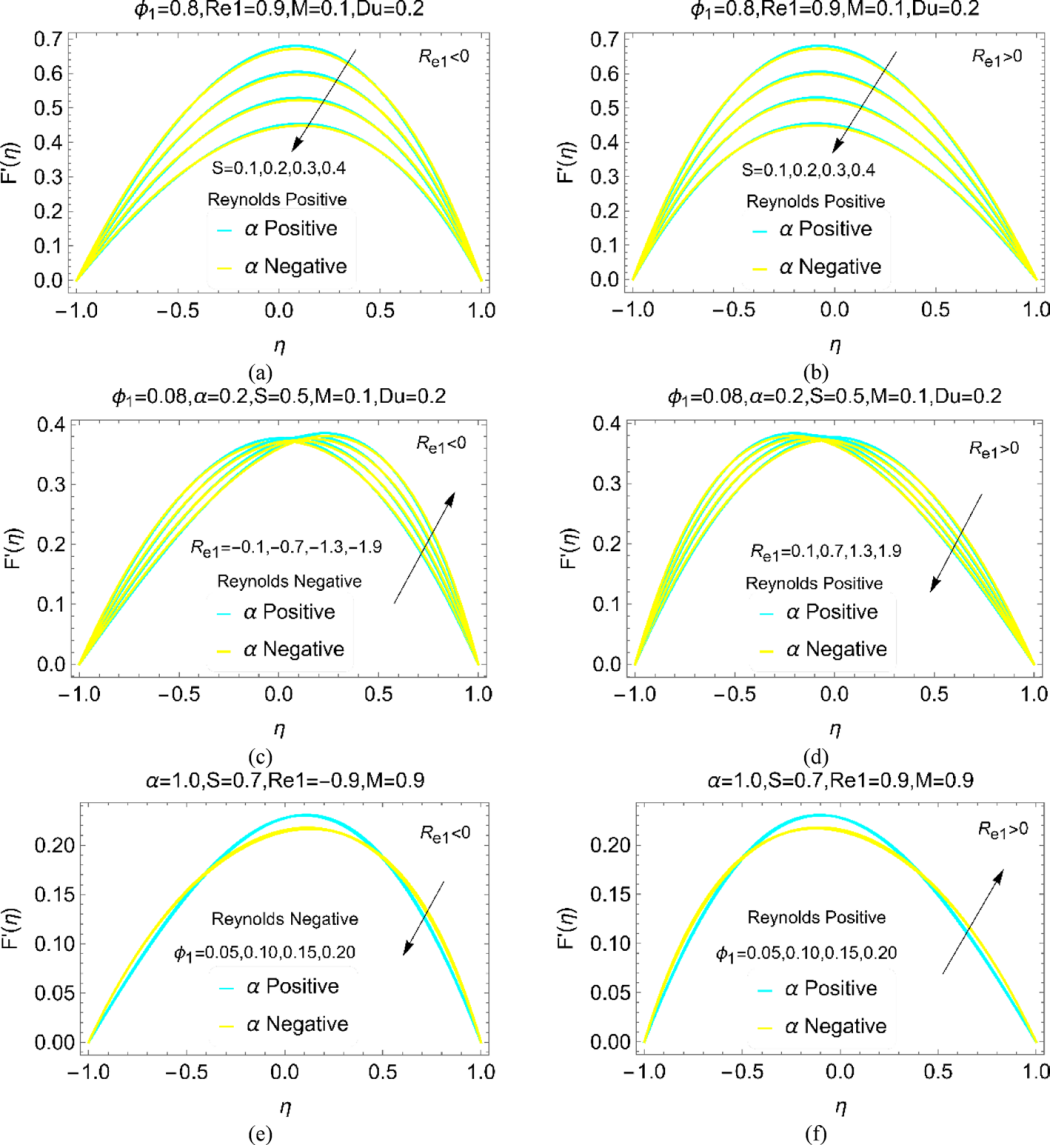
The $\:{F}^{{\prime\:}}\left(\eta\:\right)$ trends for the problem parameters

**Fig. 3:**
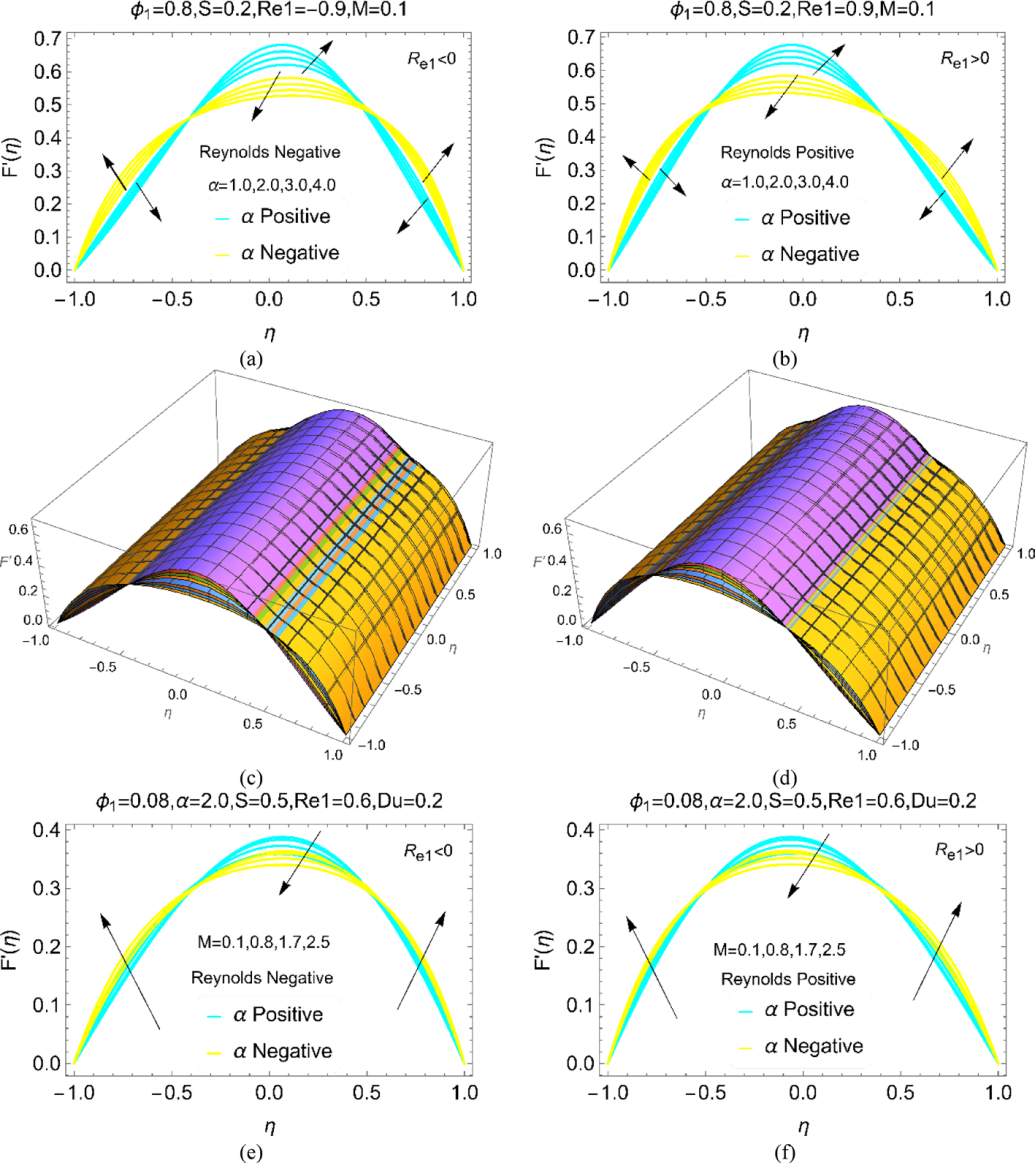
The $\:F{\prime\:}\left(\eta\:\right)$ trends for $\:\alpha\:$ and $\:M$

This section provides a deep investigation of the model constraints on the behaviour of fluid dynamics in desired domain. The varying parameters influence on the velocity is furnished in Figs. 2 and 3. The comparative results plotted to examine the importance of contraction/expansion ($\:\alpha\:$) of the walls in the presence of uniform permeation ($\:S$). The permeation of the walls hinders the fluid movement in both scnerios. Physically, the fluid drains out from the channel through pores at the surfaces. This process disturbs the fluid near the walls and reduces its momentum as a consequence the velocity declines. Expansion of the walls slowly reduces the motion in comparison with contraction case. Physically, contracting walls pressed the fluid towards the walls which lead to rapid fluid drain through the permeable surface. Further, the Reynolds number for multiple stages also affects the velocity and opposite changes are examined in the channel towards upper wall. The positive Reynolds values observed to be beneficial to regulate the fluid at low momentum which is interest in engineering and industrial applications. Moreover, adding concentration in the base fluid also declines the velocity by strengthening the viscosity.

The promising influence of expansion ($\:\alpha\:>0$) and contraction ($\:\alpha\:<0$) on the velocity of fluid are examined in Fig. 3. The expanding walls increases the motion at middle of the channel in both cases. Physically, consistent expansion of the walls gradually increases the flow area as a result the particles move freely in there and ultimately the momentum upsurges. Further, the fluid movement changes near the walls and reduces the velocity. Physically, these variations are due to adverse pressure at there which resists the fluid particles movement and the velocity decreases. On the other hand, the magnetic field also observed helpful to maintain the fluid at slow motion. This would beneficial for purification purposes in industries where the fluid flows inside the channel under uniform permeation condition.

**Fig. 4:**
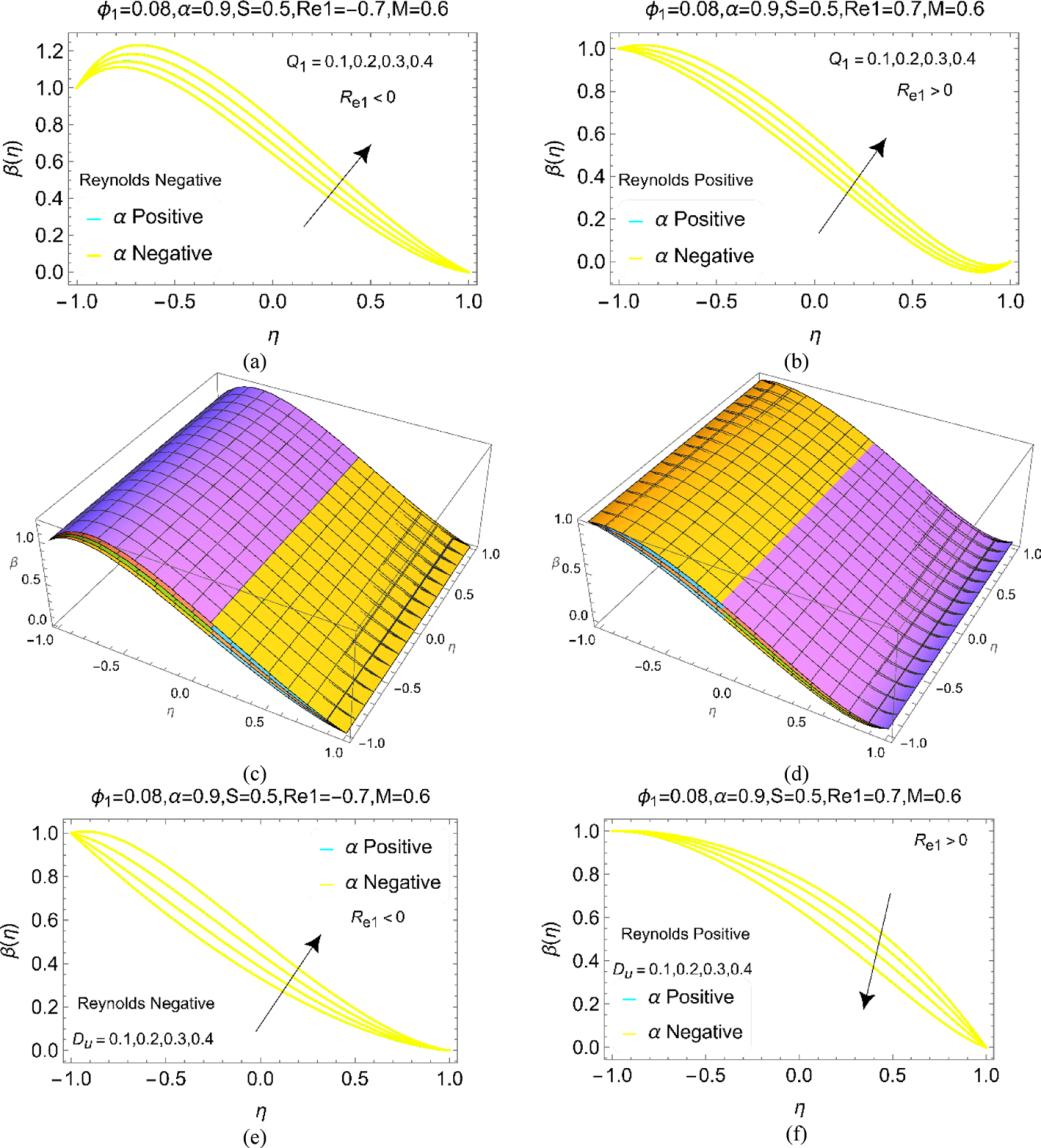
The $\:\beta\:\left(\eta\:\right)$ for the problem parameters

**Fig. 5:**
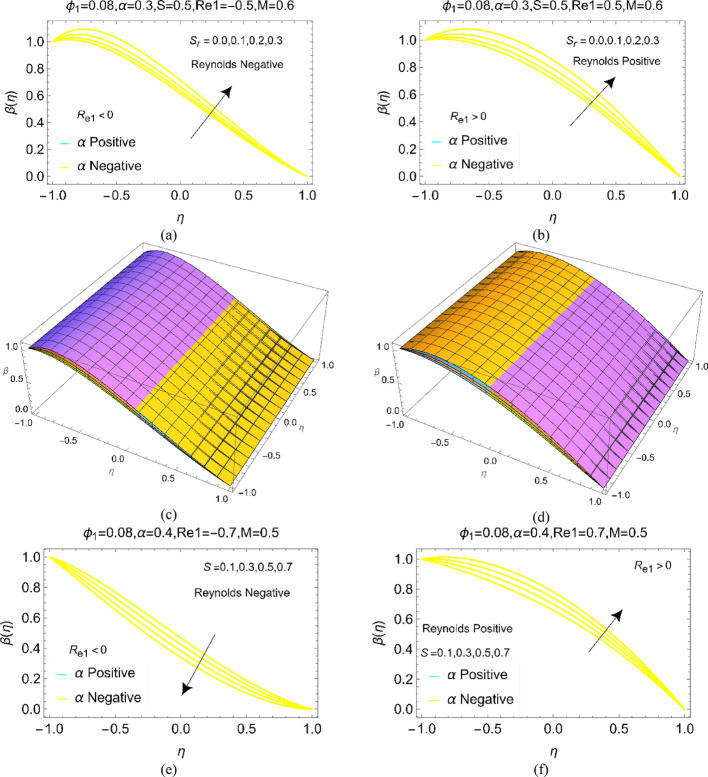
The temperature for $\:Sr$ and $\:S$

**Fig. 6:**
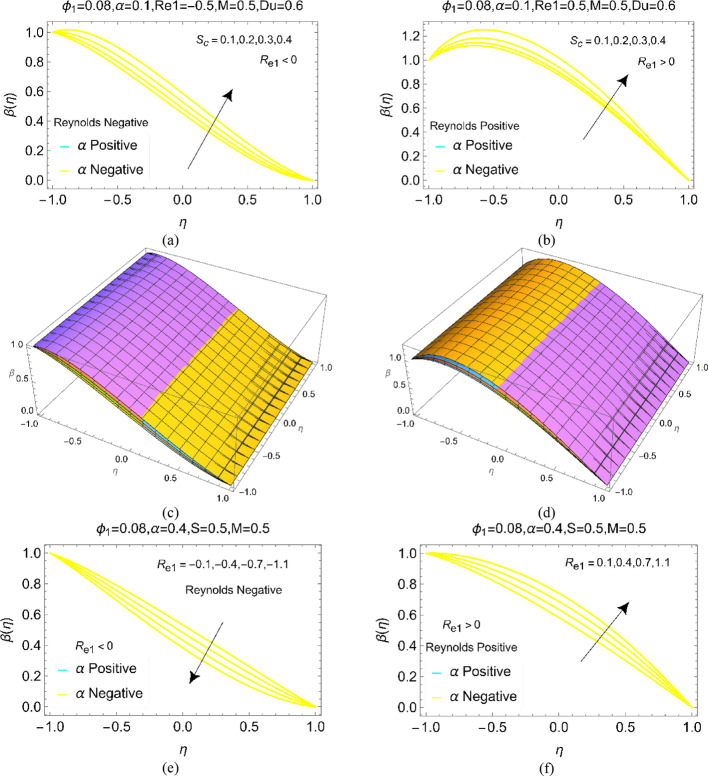
The temperature for $\:Sc$ and $\:R{e}_{1}$

Figures 4, 5 and 6 furnishing the impacts of parameters on the fluid temperature inside the channel. Figure 4 presenting the role of heat generation $\:{Q}_{1}$ and Dufour ($\:Du$) number on $\:\beta\:\left(\eta\:\right)$ in the presence of contracting and expanding boundaries. Strengthening $\:{Q}_{1}$ contributing potentially in the temperature of the nanofluid. Physically, heat generation provides extra heat energy to the fluid which make it more energetic and transmits the energy to nearby particles. This physical process then boosts the temperature of the system. Thus, adding heat generation factor in the model is highly influential for heat transfer applications in industries. Dufour number has opposite effects on the temperature based on $\:R{e}_{1}>0$ and $\:R{e}_{1}<0$. It is examined that negative permeation with increasing $\:Du$ effects is beneficial for heat transfer while positive permeation reduces it inside the channel.

Figure 5 highlights that Soret effects on the temperature and noticeable increase in observed for the both cases. Increase in the $\:Sr$ values provide considerable increase in the nanofluid temperature. Physically, it enhances the concentration gradients which promotes thermal energy and increasing the temperature. Thus, higher Soret effects added more thermal energy due to which quick thermal response is observed. Further, the permeability $\:S$ with negative Reynolds value depreciates the temperature and it enhances with positive $\:R{e}_{1}$. The Schmidt effects positively contributing in the temperature field and it would be helpful to achieve desired temperature in the nanofluidic system inside the channel with consistent permeation. Moreover, multiple stages of $\:R{e}_{1}$ for positive and negative values has inverse effects on the temperature.

**Fig. 7:**
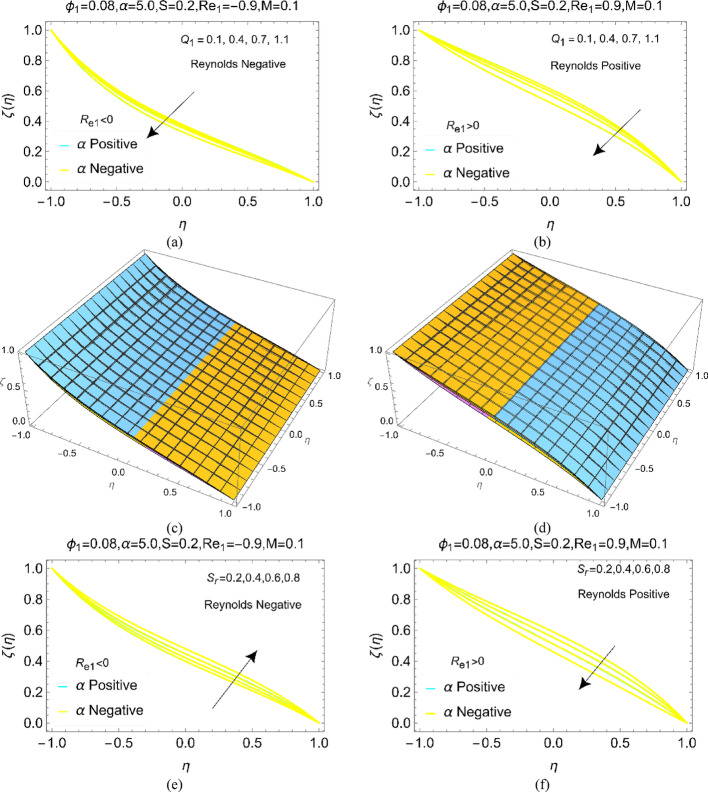
The $\:\zeta\:\left(\eta\:\right)$ for parameters

**Fig. 8:**
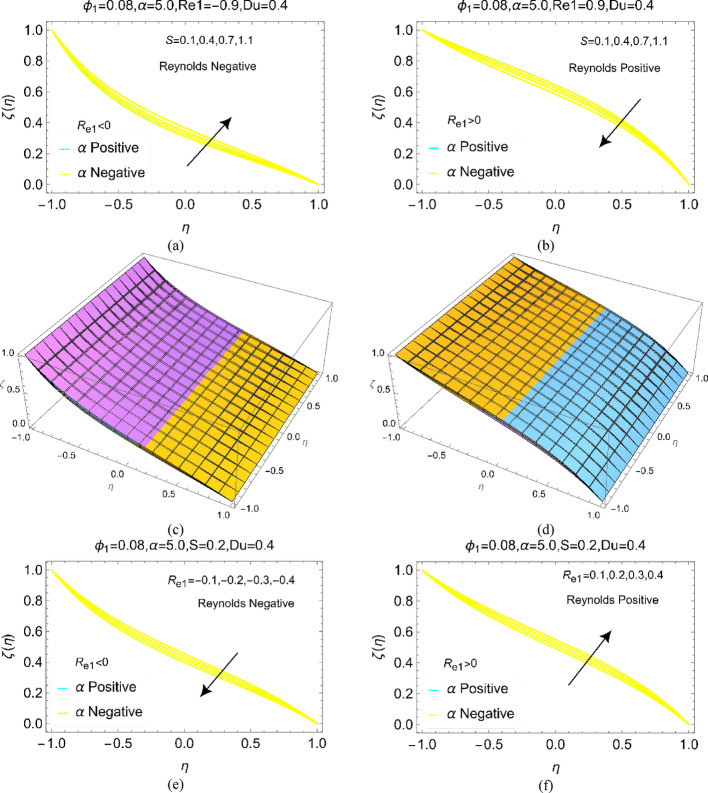
Multiple parameters impact on $\:\zeta\:\left(\eta\:\right)$

**Fig. 9:**
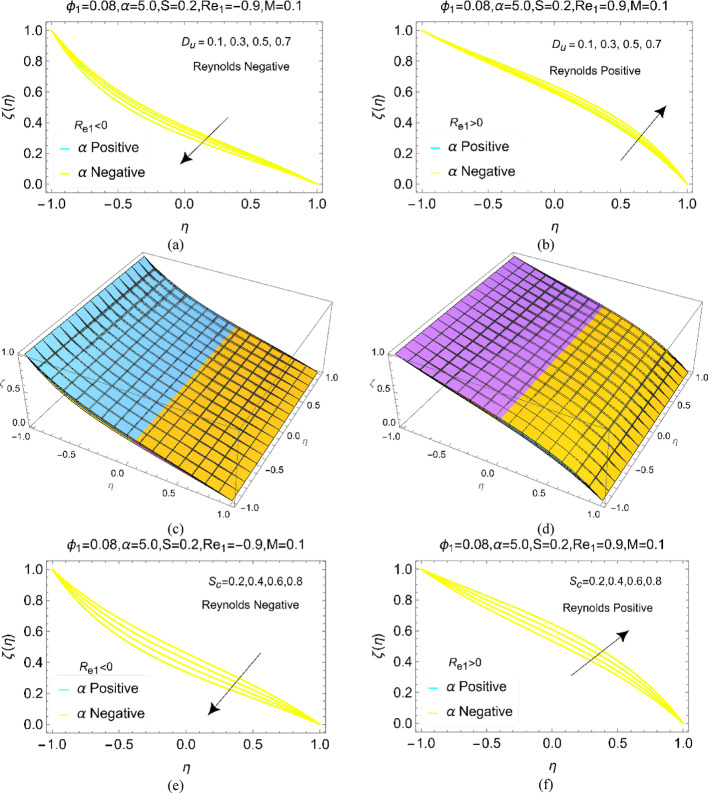
The $\:Du$ and $\:Sc$ impacts on $\:\zeta\:\left(\eta\:\right)$

Figures 7, 8, and 9 highlighting the influence of parameters on the concentration field $\:\zeta\:\left(\eta\:\right)$ in desired domain of expanding and contracting channel. The heat generation controls the concentration of the nanofluid with both positive and negative Reynolds situations. The Soret and permeable parameters have considerable effects on $\:\zeta\:\left(\eta\:\right)$. It enhances the concentration while Reynolds parameter attains positive value. However, it drops the concentration in the case of negative value. The Schmidt number has prominent effects on the concentration field which shows its applicability under negative $\:R{e}_{1}$ to control and positive $\:R{e}_{1}$ to improves the concentration in the channel with microorganisms.

**Fig. 10:**
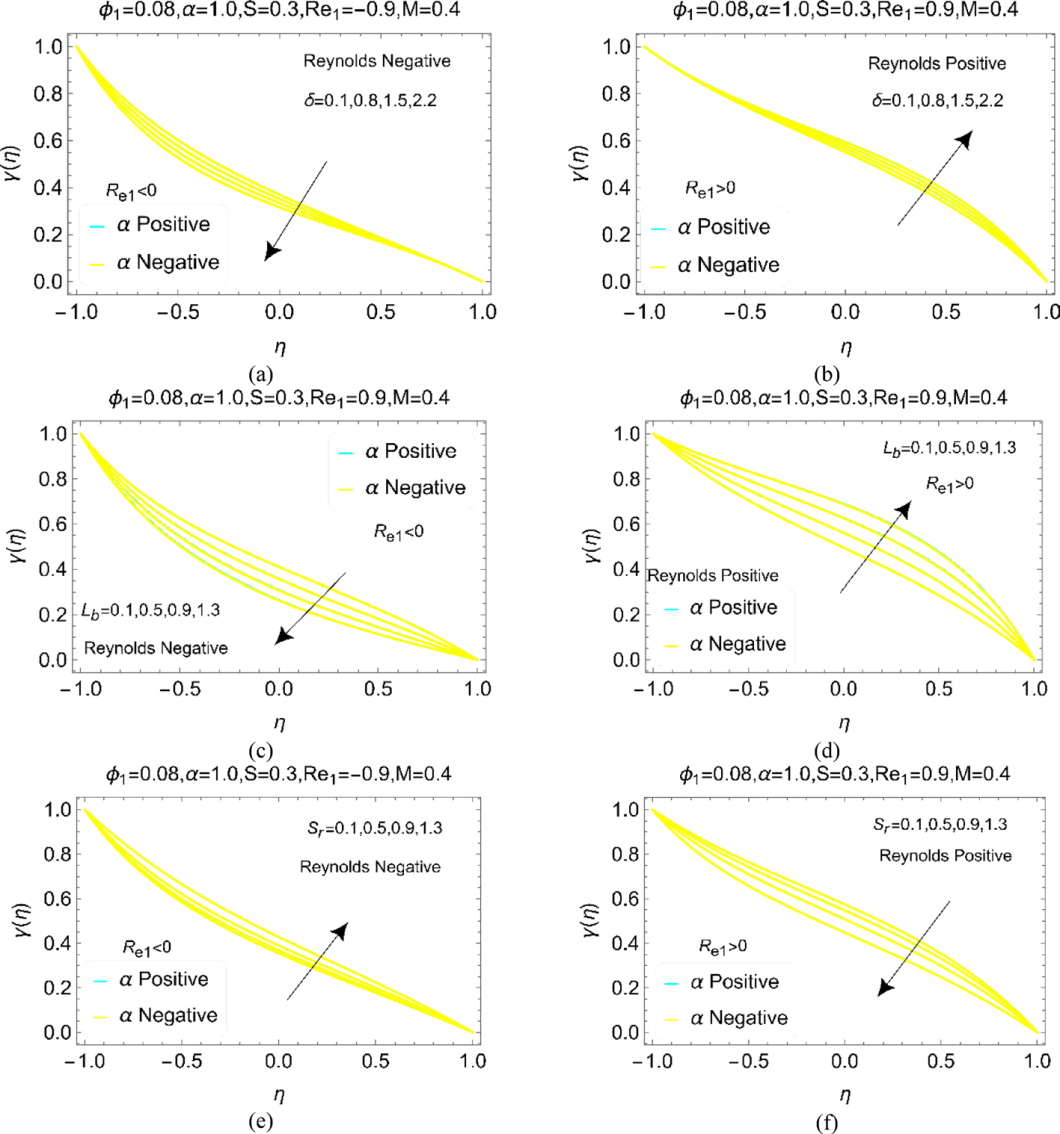
The $\:\gamma\:\left(\eta\:\right)$ changes for multiple quantities

**Fig. 11:**
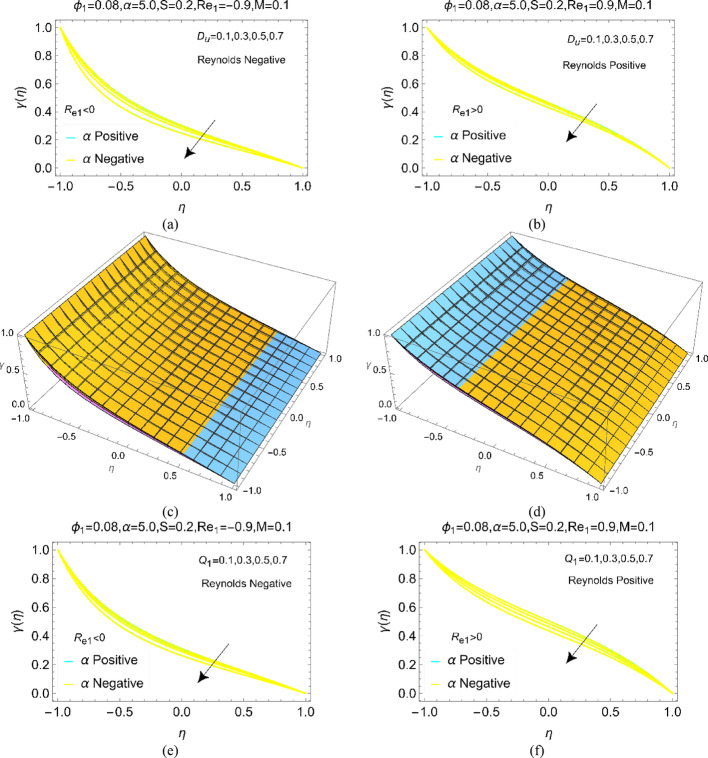
The changes in $\:\gamma\:\left(\eta\:\right)$ for $\:Du$ and $\:{Q}_{1}$

**Fig. 12:**
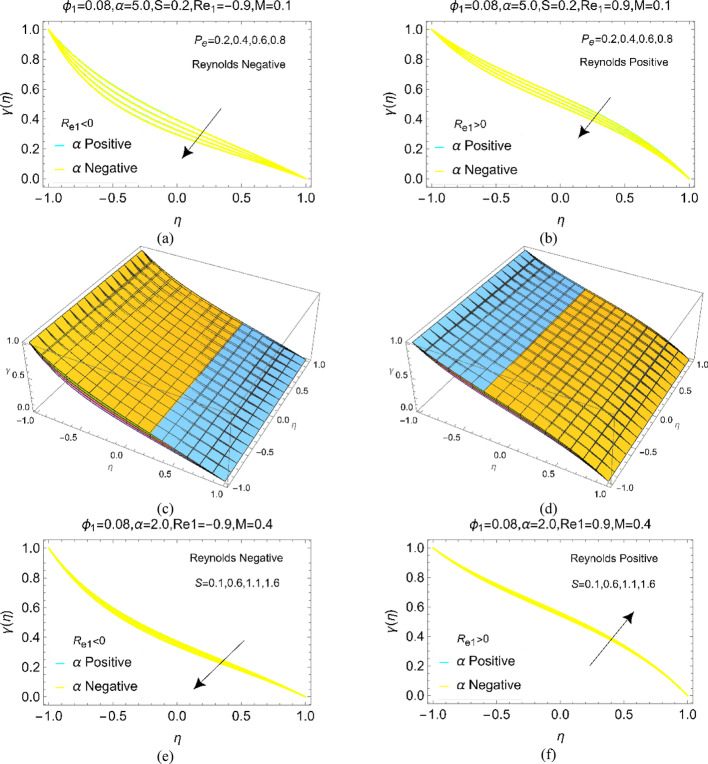
Impacts of $\:Pe$ and $\:S$ on $\:\gamma\:\left(\eta\:\right)$

**Fig. 13:**
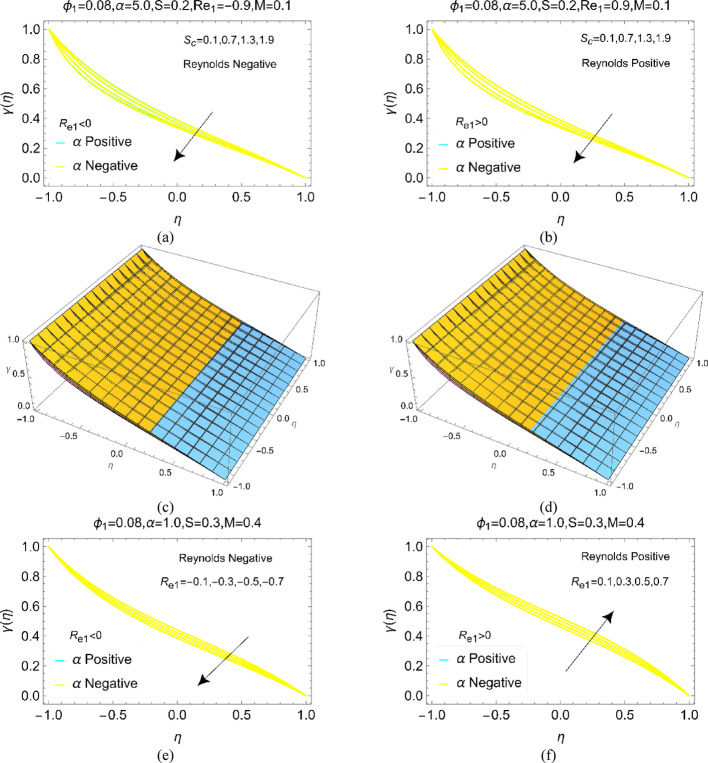
The $\:Sc$ and $\:R{e}_{1}$ impacts on $\:\gamma\:\left(\eta\:\right)$

Figure 10c, d depicts the impact of Lewis parameter $\:{L}_{b}$ on density motile of microorganism $\:\gamma\:\left(\eta\:\right)$. upon gamma profile. It is noted that $\:\delta\:$ and $\:{L}_{b}$ promotes density motile filed for $\:R{e}_{1}>0$ while it opposes for $\:R{e}_{1}<0$. The $\:{L}_{b}$ has prominent changes in $\:\gamma\:\left(\eta\:\right)$ than $\:\delta\:$ in both scnerios. The Sort number enhances the motile profile for negative Reynolds and upsurges it for positive Reynolds case. The Dufour number has minimal effects on $\:\gamma\:\left(\eta\:\right)$ than Soret number in expanding and contracting channel. Further, the heat generation $\:{Q}_{1}$, Peclet $\:Pe$ and Schmidt $\:Sc$ numbers resist the density motile organisms in both physical scnerios. Thus, augmenting these parameters would help to control the density motile of microspecies in the working nanofluid. These results are furnished in Figs. 11, 12, and 13, respectively. Thus, these physical quantities have remarkable impacts on $\:\gamma\:\left(\eta\:\right)$ which plays key role in biomedical engineering for handling the movement of microspecies.

It is examined from the computation given in Table 1 that the shear drag rises for expansion case by influencing the factor $\:{\phi\:}_{1}$. It is depreciated for absorptive factor fluctuates with further factors kept constant. The measured disclose that the magnetic number is fundamental key to acquire extreme skin friction. Table 2 presenting the computational values for Nusselt numbers associated to the model quantities. The intense analysis of the values indicates that increasing the permeable variable from 2.0 to 8.0% is admirable achieve the preferred thermal gradient. Conversely, The Soret factor condenses the significance of Nusselt number.

**Table 1:** The skin friction for the model

$\:{\boldsymbol{\phi\:}}_{1}$	$\:\boldsymbol{a}$	S	$\:{\boldsymbol{R}}_{\boldsymbol{e}1}$	M	$\:\boldsymbol{F}^{\boldsymbol{{\prime\:}}\boldsymbol{{\prime\:}}}(-1)$		$\:\left|{\boldsymbol{F}}^{\boldsymbol{{\prime\:}}\boldsymbol{{\prime\:}}}\left(1\right)\right|$	
	$\:3.0$	$\:0.2$	$\:0.9$	$\:0.1$	Expansion	Contraction	Expansion	Contraction
$\:0.01$					$\:0.9057$	$\:1.9017$	$\:0.6762$	$\:1.3982$
$\:0.02$					$\:0.9058$	$\:1.9010$	$\:0.6764$	$\:1.3979$
$\:0.03$					$\:0.9061$	$\:1.8994$	$\:0.6768$	$\:1.3973$
$\:0.04$					$\:0.9064$	$\:1.8970$	$\:0.6774$	$\:1.3964$
$\:0.06$	$\:1.0$				$\:1.1458$	$\:1.4689$	$\:0.8327$	$\:1.0615$
	$\:2.0$				$\:1.0173$	$\:1.6644$	$\:0.7491$	$\:1.2123$
	$\:3.0$				$\:0.9093$	$\:1.8805$	$\:0.6819$	$\:1.3900$
	$\:4.0$				$\:0.8197$	$\:2.1170$	$\:0.6284$	$\:1.5955$
	$\:0.3$	$\:0.2$			$\:0.9076$	$\:1.8901$	$\:0.6793$	$\:1.3937$
		$\:0.4$			$\:0.6993$	$\:1.4600$	$\:0.4954$	$\:1.0240$
		$\:0.6$			$\:0.4796$	$\:1.0096$	$\:0.3209$	$\:0.6686$
		$\:0.8$			$\:0.2470$	$\:0.5220$	$\:0.1558$	$\:0.3273$
		$\:0.2$	$\:0.1$		$\:0.78703$	$\:1.6863$	$\:0.7599$	$\:1.6279$
			$\:0.3$		$\:0.8167$	$\:1.7366$	$\:0.7368$	$\:1.5638$
			$\:0.5$		$\:0.8467$	$\:1.7874$	$\:0.7157$	$\:1.5035$
			$\:0.7$		$\:0.8770$	$\:1.8386$	$\:0.6966$	$\:1.4469$
			$\:0.9$	$\:0.1$	$\:0.9076$	$\:1.8901$	$\:0.6793$	$\:1.3937$
				$\:0.3$	$\:0.9114$	$\:1.8937$	$\:0.6836$	$\:1.3997$
				$\:0.5$	$\:0.9191$	$\:1.9010$	$\:0.6922$	$\:1.4116$
				$\:0.7$	$\:0.9304$	$\:1.9120$	$\:0.7050$	$\:1.4292$

**Table 2:** Nusselt number against the model quantities

$\:{\boldsymbol{\phi\:}}_{1}$	$\:\boldsymbol{\alpha\:}$	S	$\:{\boldsymbol{R}}_{\boldsymbol{e}1}$	M	$\:{\boldsymbol{D}}_{\boldsymbol{u}}$	$\:{\boldsymbol{Q}}_{1}$	$\:{\boldsymbol{S}}_{\boldsymbol{r}}$	$\:{\boldsymbol{S}}_{\boldsymbol{c}}$	$\:\left|{-\boldsymbol{\beta\:}}^{\boldsymbol{{\prime\:}}}\left(-1\right)\right|$		$\:\left|-\boldsymbol{\beta\:}^{\boldsymbol{{\prime\:}}}\left(1\right)\right|$	
	$\:2.0$	$\:0.2$	$\:0.9$	0.5	0.5	0.3	0.4	0.4	Expansion	Contraction	Expansion	Contraction
0.01									$\:0.0509$	$\:0.0480$	$\:1.4957$	$\:1.4922$
0.02									$\:0.0528$	$\:0.0470$	$\:1.4968$	$\:1.4898$
$\:0.03$									$\:0.0547$	$\:0.0464$	$\:1.4976$	$\:1.4800$
$\:0.04$									$\:0.0568$	$\:0.0461$	$\:1.4981$	$\:1.4839$
$\:0.06$	$\:1.0$								$\:0.0509$	$\:0.0480$	$\:1.4957$	$\:1.4922$
	$\:2.0$								$\:0.0528$	$\:0.0470$	$\:1.4968$	$\:1.4922$
	$\:3.0$								$\:0.0547$	$\:0.0464$	$\:1.4976$	$\:1.4821$
	$\:4.0$								$\:0.0568$	$\:0.0461$	$\:1.4981$	$\:1.4839$
	$\:2.0$	$\:1.0$							$\:0.7342$	$\:0.7342$	$\:1.9524$	$\:1.9520$
		$\:2.0$							$\:1.6076$	$\:1.6061$	$\:2.3917$	$\:2.3991$
		$\:3.0$							$\:2.4182$	$\:2.4206$	$\:2.7745$	$\:2.7945$
		$\:4.0$							$\:3.1592$	$\:3.1746$	$\:3.0978$	$\:3.1381$
		$\:0.2$	$\:0.2$						$\:0.3707$	$\:0.3699$	$\:0.9571$	$\:0.9563$
			$\:0.3$						$\:0.2715$	$\:0.2700$	$\:1.1306$	$\:1.1289$
			$\:0.4$						$\:0.1677$	$\:0.1656$	$\:1.1306$	$\:1.3013$
			$\:0.5$						$\:0.0595$	$\:0.0567$	$\:1.4771$	$\:1.4737$
			$\:0.9$	$\:0.1$					$\:0.6438$	$\:0.6441$	$\:1.9055$	$\:1.9050$
				$\:0.4$					$\:0.6437$	$\:0.6440$	$\:1.9054$	$\:1.9049$
				$\:0.7$					$\:0.6436$	$\:0.6439$	$\:1.9053$	$\:1.9047$
				$\:1.0$					$\:0.6434$	$\:0.6437$	$\:1.9049$	$\:1.9040$
				$\:0.5$	$\:0.1$				$\:0.6403$	$\:0.6406$	$\:2.0474$	$\:2.0469$
					$\:0.3$				$\:0.6461$	$\:0.6465$	$\:1.7511$	$\:1.7506$
					$\:0.5$				$\:0.6481$	$\:0.6484$	$\:1.4010$	$\:1.4003$
					$\:0.7$				$\:0.6441$	$\:0.6445$	$\:0.9820$	$\:0.9813$
					$\:0.5$	0.2			$\:0.6481$	$\:0.6484$	$\:1.4010$	$\:1.4003$
					$\:0.5$	0.4			$\:1.0177$	$\:1.0180$	$\:1.6950$	$\:1.6943$
						0.6			$\:1.4728$	$\:1.4731$	$\:2.0779$	$\:2.0772$
						0.8			$\:2.0545$	$\:2.0549$	$\:2.5938$	$\:2.5930$
						0.3	0.1		$\:0.6599$	$\:0.6602$	$\:1.3748$	$\:1.3742$
							0.3		$\:1.0342$	$\:1.0345$	$\:1.7619$	$\:1.7612$
							0.5		$\:1.6920$	$\:1.6925$	$\:2.5360$	$\:2.5351$
							0.7		$\:3.0889$	$\:3.0896$	$\:4.433$	$\:4.4322$
							0.4	$\:0.1$	$\:1.0544$	$\:1.0547$	$\:1.9061$	$\:1.9055$
								$\:0.2$	$\:0.9994$	$\:0.9997$	$\:1.8310$	$\:1.8305$
								$\:0.3$	$\:0.9425$	$\:0.9428$	$\:1.7457$	$\:1.7451$
								$\:0.4$	$\:0.8839$	$\:0.8843$	$\:1.6488$	$\:1.6482$

## Conclusions

Indepth investigation of hybrid nanofluid suspended by microorganism is conducted and treated through analytical scheme. The study reveals that.

- The velocity significantly controlled for increasing $\:S=0.1$ to $\:S=0.4$ and optimum declines is observed near the central portion of the channel and negative permeation $\:{R}_{e1}<0$ enhances the velocity in the upper part.
- The varying concentration (from 0.05 to 0.20) of hybrid nanomaterial and magnetic field reduces the fluid motion.
- The heat generation $\:{Q}_{1}$ improves the temperature in expanding and contracting walls while Dufour number declines it for $\:{R}_{e1}>0$.
- The Soret number help to enhance the density motile profile of microorganisms for positive permeation while it drops it for negative permeation.
- Expansion of the walls reduces the skin friction against the parameters while contraction case has higher shear drag magnitude at both the walls.
- The magnitude of heat transfer rate improves from 0.0509 to 0.0568 in expansion, 1.4957 to 1.4981 (expansion case), while it drops from 0.0480 to 0.0461, and 1.4922 to 1.4839 (contracting case).
- Soret number in the range of 0.1 to 0.4 controls the magnitude of heat transfer rate at both the surfaces under expanding and contraction conditions. Thus, it would be beneficial to maintain the heat in the fluidic system.

The model has further openings by considering the variety of nanomaterials of hybrid, Corcione model [48], ternary and tetra types [49] with influence of chemical reaction, activation energy, and nonlinear radiations using AI drive approaches [50]. Moreover, the problem is extendable for non-Newtonian fluids coupled with alloys and metallic nanomaterial. Also, extending the boundaries towards convective and slip effects are also potential directions in the future.

## Data Availability

This work has no associated data.

## Nomenclature

$$ \mathop u\limits^{\sim } ,\mathop v\limits^{\sim } $$: Velocity components (m/s)
$${{\tilde{P}}}$$: Pressure (Pa)
σ_nf_: Electrical conductivity (S/m)
k_nf_: Thermal conductivity (W/mK)
t: Time (m/s)
$$ \tilde{T} $$: Temperature (K)
μ_nf_: Dynamic viscosity (Pa.s)
h: Height (m)
η: Dimensionless variable
F': Dimensionless velocity
β: Dimensionless temperature
$$ \zeta $$: Concentration
γ: Density motile
R_e1_: Reynolds number
M: Hartmann number
Pr: Prandtl number
Du: Dufour number
Q_1_: Heat generation number
Sr: Soret number
Sc: Schmidt number
Pe: Peclet number
